# Response to: Olry de Labry Lima A et al., Cost-effectiveness of lenalidomide maintenance in patients with multiple myeloma who have undergone autologous transplant hematopoietic progenitor cells

**DOI:** 10.1038/s41409-019-0749-0

**Published:** 2019-11-21

**Authors:** Sujith Dhanasiri

**Affiliations:** Celgene International Sarl, Boudry, Switzerland

**Keywords:** Myeloma, Quality of life

## To the Editor:

Lenalidomide maintenance therapy is recommended for patients with newly diagnosed multiple myeloma (MM) post autologous stem cell transplant (ASCT) by current treatment guidelines such as the ESMO guidelines [1]. Many countries have approved the product for the indication, and offer reimbursement for the treatment, based on a substantial body of evidence indicating that, compared with no maintenance therapy, lenalidomide provides clinically meaningful and significant improvements in progression-free survival, overall survival, and health-related quality of life (HRQoL) [2–8].

In addition to the safety and efficacy of a product, the cost-effectiveness and budget impact of lenalidomide maintenance therapy are key considerations when making treatment decisions, and it is important that such analyses are performed using robust methodology and based on accurate clinical data and assumptions. We are thus concerned to note two important inaccuracies in the cost-effectiveness analysis recently published in *Bone Marrow Transplantation* by Antonio Olry de Labry Lima et al. in the article entitled “Cost-effectiveness of lenalidomide maintenance in patients with MM who have undergone autologous transplant of hematopoietic progenitor cells”, namely assuming a time horizon of 10 years in the base case, and reporting a total drug cost for lenalidomide maintenance therapy of EUR 535,407.

First, trials of ASCT in MM report an average patient age of 55–59 years and 64% of patients included in the IFM trial were aged ≤59 years [2, 3, 5]. Thus, the time horizon of 10 years used in the base case for Olry de Labry Lima’s analysis to reflect the maximum remaining life of patients is too short to capture the full costs of treatment post ASCT, including the costs of later lines of therapy for patients relapsing post ASCT. Indeed, according to the data presented in Fig. 1 of the paper by Olry de Labry Lima et al., none of the curves show full occupancy for death health state at 10 years, as would be expected after a true lifetime horizon. Furthermore, results for a sensitivity analysis using a 20-year time horizon resulted in substantial reductions to the incremental cost-effectiveness ratio (ICER). Together these results suggest that a 10-year time horizon does not capture all relevant outcomes for patients post ASCT.

Second, the reported total cost of lenalidomide maintenance therapy appears to assume a total treatment duration of 65 months (based on their stated monthly cost of EUR 8175), whereas trials report a median duration of lenalidomide therapy of 25–35 months [6]. Both assumptions inflate the ICER for lenalidomide maintenance therapy and suggest that this highly effective treatment is not cost-effective.

Various scientific publications have shown that lenalidomide delays disease progression, increases the duration of disease-free periods, and delays progression to later-line regimens involving novel combination therapies without detriment to quality of life [9–11]. These effects can be expected to result in cost savings compared with no maintenance therapy. Finding lenalidomide maintenance therapy to have an ICER of more than EUR 250,000/quality-adjusted life year (QALY) compared with no maintenance therapy, as suggested by Olry de Labry Lima’s results, is therefore counterintuitive. In contrast, a cost-effectiveness analysis from the Dutch national health service perspective reported an ICER of approximately EUR 30,000/QALY for lenalidomide maintenance therapy [12].

For economic evaluations to be used as part of treatment decision-making, it is important that they provide an accurate evaluation of cost-effectiveness, since concluding a treatment is not cost-effective may have implications for patient access to life-prolonging therapies that improve their HRQoL. We suggest that Olry de Labry Lima’s analysis is not an accurate economic evaluation of lenalidomide maintenance therapy, based on the points highlighted above.
